# A Structural Perspective of Reps from CRESS-DNA Viruses and Their Bacterial Plasmid Homologues

**DOI:** 10.3390/v14010037

**Published:** 2021-12-25

**Authors:** Elvira Tarasova, Reza Khayat

**Affiliations:** Department of Chemistry and Biochemistry, City College of New York, New York, NY 10031, USA; elviratarasovachem@gmail.com

**Keywords:** CRESS-DNA, pCRESS-DNA, replicase, rolling circle replication, structure, endonuclease, ATPase, helicase

## Abstract

Rolling circle replication (RCR) is ubiquitously used by cellular and viral systems for genome and plasmid replication. While the molecular mechanism of RCR has been described, the structural mechanism is desperately lacking. Circular-rep encoded single stranded DNA (CRESS-DNA) viruses employ a viral encoded replicase (Rep) to initiate RCR. The recently identified prokaryotic homologues of Reps may also be responsible for initiating RCR. Reps are composed of an endonuclease, oligomerization, and ATPase domain. Recent structural studies have provided structures for all these domains such that an overall mechanism of RCR initiation can begin to be synthesized. However, structures of Rep in complex with its various DNA substrates and/or ligands are lacking. Here we provide a 3D bioinformatic review of the current structural information available for Reps. We combine an excess of 1590 sequences with experimental and predicted structural data from 22 CRESS-DNA groups to identify similarities and differences between Reps that lead to potentially important functional sites. Experimental studies of these sites may shed light on how Reps execute their functions. Furthermore, we identify Rep-substrate or Rep-ligand structures that are urgently needed to better understand the structural mechanism of RCR.

## 1. Introduction

Rolling-circle replication (RCR) was introduced by Gilbert and Dressler more than half a century ago to describe the molecular mechanism of genome replication by the bacteriophage φX174 [1]. Sometime thereafter, RCR was also used to describe replication of bacterial plasmid pT181 [2,3]. Since then, the use of RCR by viral/phage, prokaryotic plasmids, and eukaryotic transposons has become well documented [4,5,6,7,8,9,10,11,12,13,14]. One such system is the replication of viruses with circular single stranded DNA (ssDNA) genomes. While these studies have provided the molecular mechanism describing RCR, structural detail is desperately lacking. RCR begins with an initiator protein binding to a sequence specific origin of replication (*ori*) located on the double stranded DNA (dsDNA). According to the number of functional domains they possess, initiator proteins can be categorized into two groups: (1) the pT181-like group, and (2) the circular-rep encoded single stranded DNA (CRESS-DNA) group. The pT181-like plasmids are promiscuous and naturally occurring plasmids involved in antibiotic resistance [15]. CRESS-DNA viruses, members of pylum *Cressdnaviricota*, were first described by Rosario et al. to include a group of viruses that possessed circular ssDNA genomes and encode an enzyme responsible for initiating genome replication [16,17]. These viruses are highly prevalent and infect species from all domains of life [6,14,16,18,19]. The initiator proteins for pT181-like prokaryotic plasmids are referred to as relaxases (e.g., RepC). Relaxases consist of two domains: (1) an endonuclease domain (ED), and (2) and oligomerization domain (OD). The initiator proteins for CRESS-DNA viruses are referred to as Replicases (Reps). Reps consists of at least three domains: (1) an endonuclease domain (ED) at the N-terminus, (2) an oligomerization domain (OD) in the middle, and (3) an ATPase domain (AD) at the C-terminus [14,20]. Relaxes and Reps bind to *ori* and deform the dsDNA to form a cruciform structure. The exposed nonanucleotide loop of the (+) ssDNA is the substrate for hydrolysis by ED. ED is a member of the HUH (His-hydrophobic-His motif) endonuclease group. Members of this group cut ssDNA at sequence specific sites. Cutting the ssDNA generates a 3′-OH that is used for leading strand DNA synthesis. ED uses a Tyr nucleophile for DNA hydrolysis, and the Tyr becomes covalently attached to the 5′-PO4 of the (+) ssDNA. Depending on the system, strand separation and DNA polymerization are accomplished by cellular, viral, or phage encoded helicase and polymerase. Replication continues beyond the start site, defined by the ED cut site, such that the additional nucleotides allow for a stem-loop structure to be formed for processing again by ED. ED hydrolyses the freshly formed stem-loop structure to liberate the ssDNA bound to Rep from dsDNA. The two ends of the replicant genome are then ligated by ED to generate the circular ssDNA [14]. Crystal structures of Relaxases and Reps in complex with their ssDNA substrate define how these proteins attain sequence specificity; however, there is lack of structural information on *ori* recognition, cruciform formation, and ssDNA ligation. The OD serves to oligomerize Relaxases and Reps into hexamers and acts as the conduit for ssDNA translocation in Reps [20,21]. The AD of Reps are members of the Super Family 3 (SF3), which belong to the ATPases Associated with diverse cellular Activities (AAA+) families [20,22]. The AD is responsible for the helicase activity of Rep. Reps are not true replicases, as they initiate replication but are not responsible for DNA elongation.

Bioinformatic approaches studying the phylogenetic relationship between Reps have identified conserved motifs that may serve important functional roles [16,19,23,24,25,26,27]. A study by Kazlauskas et al. demonstrated that prokaryotes also use extrachromosomal and integrated plasmids to code for Rep like proteins. They named this group pCRESS Reps and revised the CRESS name to CRESSV to distinguish them as viral [19]. In total, they identified 23 groups of Reps. Up until recently, structural information for CRESS-DNA Reps existed only for the ED. Porcine circovirus type 2 (PCV2) may be regarded as the prototypical member of CRESS-DNA viruses as it is the most commonly studied member. We recently determined the structure of the PCV2 Rep in complex with ssDNA and adenosine diphosphate (ADP) [20]. The structure demonstrated a lock washer-like hexamer whose central channel was occupied by a hexanucleotide ssDNA. Six ADs spiraled around the ssDNA like a staircase and made limited interactions with one another. The AD at the top and bottom of the staircase made minimal interactions with the ssDNA. ADP was observed to bind four of the six AD–AD interfaces, with the interfaces for the two ADs near the bottom of the staircase being nucleotide free. We were unable to resolve density associated with the six ED, presumably due to their flexibility. Furthermore, we demonstrated that PCV2 Rep exhibited basal NTPase activity. In conjunction with the literature describing the structure of PCV2 ED and PCV2 ED bound to ssDNA, there are now atomic coordinates describing the full-length PCV2 Rep [20,28,29,30]. Our PCV2 Rep structure inspired us to ask: how conserved are the primary and tertiary structures of Reps, how conserved are the functional centers of Reps, and are there distinctions between Reps that may shed light on their functional activity?

To answer these questions, we combined sequence analysis with the structural data available on PCV2 Rep. We demonstrate that all 22 groups share the endonuclease, oligomerization, and ATPase domains. This suggests that all Reps are likely to share a common ancestor. We further show that the ED, OD, and AD exhibit independent levels of diversity in sequence length and identity within and between groups. This suggests that each domain is independently under evolutionary pressure. We also demonstrate that the extended (more than 50 amino acids) termini of some groups are predicted to possess structures that may be important for the regulation of these Reps. Finally, we calculated the conservation index for each amino acid using sequence alignments and mapped these values onto the predicted structures to attain positional conservation heat maps. The heat maps identify amino acids that may be functionally important.

## 2. Materials and Methods

The sequences deposited by Kazlauskas et al. were attained from the supplementary section of the publication [19]. Sequences from each group were individually submitted to BlastP to retrieve sequences with more than 30% sequence identity and 80% coverage [31]. The attained sequences for each group were combined and submitted to the CD-Hit server to elect a single representative for sequences with more than 90% sequence identity [32]. Sequences for each group were aligned using the default parameters of Clustal Omega [33]. Sequence identity between all members of a group was attained using Clustal Omega. WebLogo images were generated using the WebLogo 3 server [34].

The CD-Hit server was also used to identify a single representative for each group. This sequence was submitted to the Robetta server for structure prediction using the TrRosetta algorithm [35]. Structure alignments, root mean square deviation (rmsd) and TM-scores were calculated using TM-align [36]. Structures were aligned onto the crystal structure of PCV2 ED (PDB entry 5XOR) and cryo-EM structure of PCV2 OD-AD (PDB entry 7LAR) [20,28]. Aligned sequences were mapped onto the structures using the Multialign Viewer tool of UCSF Chimera [37]. Heat maps were generated using the default parameters of AL2CO and render by attribute tools of UCSF Chimera [38]. Sequences for domains were extracted using the Jalview 2 software [39]. Domain boundaries were manually defined according to the predicted domains. These are N-terminus: sequence prior to β1 of ED; ED: β1 to end of ED β5; ED-OD linker: end of ED β5 to beginning of OD αa; OD: end of ED β5 to beginning of AD β1; AD: AD β1 to end of AD β5; C-terminus: end of AD β5 to end of sequence. The domain sequences were submitted to the Clustal Omega server with no alignment requested. The percent identity matrix generated by the server served as the source for the reported sequence conservation. Structures for the N- and C-termini were predicted using the procedure described above [35]. Box scatter plots and Analysis of Variance (ANOVA) with Tukey test were performed with Microcal Origin 2016.

## 3. Results

### 3.1. Primary Structure Diversity among Reps

**Figure 1:**
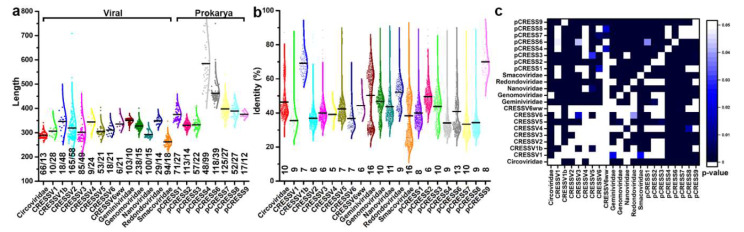
Primary protein structure comparison of CRESS-DNA and pCRESS-DNA Reps: (**a**) Box scatter plot of the sequence lengths from 1595 unique sequences deposited into GenBank. The numbers at bottom identify number of sequences compared/standard deviation in lengths. The Reps have been divided into two groups: those that are of viral origin and those that are of prokarya origin; (**b**) Box scatter plot of sequence identity within each group. Number at bottom represent standard deviation of data; (**c**) Heat map of *p*-values calculated using Analysis of Variance Tukey test. Top left compares sequence identity and bottom right compares sequence lengths.

We begin by asking, what is the diversity of primary structure within each of the 23 groups identified by Kazlauskas et al., and do the groups demonstrate comparable diversity? We attained a total of 1595 unique sequences, and each sequence could be assigned to one of the 22 groups defined by Kazlauskas et al. (see methods and materials section). We note that of these groups, six have been designated as families by the International Committee of Virus Taxonomy. We set an upper threshold of 90% sequence identity to remove redundant entries. We exclude sequences from pCRESS5 because all these sequences share more than 90% sequence identity. To begin answering our question we determined the diversity of sequence lengths within each group (Figure 1a,c). The *Smacoviridae* exhibit the shortest length with a mean of 262 amino acids, and the pCRESS4 exhibits the longest length with a mean of 584 amino acids. The standard deviation, reported on the graphs, for each group can be used to assess the diversity of the data, with larger values representing greater diversity. *Geminiviridae*, pCRESS9, and *Circoviridae* exhibit a narrow range of Rep lengths, whereas pCRESS4, CRESSV3, and CRESSV2 exhibit a broad range of lengths. To determine if the observed difference of means between the groups was statistically significant, we performed an all-vs-all Analysis of Variance (ANOVA) Tukey test (Figure 1c). The *p*-values clearly demonstrate that significant differences exist between the lengths of Rep from different groups. The difference in Rep length may be indicative of differences in regulatory mechanisms, binding to nucleic acid (e.g., recognition of origin of replication), and/or nucleic acid translocation.

We assessed the diversity of sequence identity within and between groups (Figure 1b,c). Members of pCRESS9 (mean value of 70%) and CRESSV1b (mean value of 69%) share the highest sequence identity, whereas members of pCRESS7 (mean value of 33%) and CRESSV1 (mean value of 35%) share the lowest sequence identity. All groups demonstrate a broad range of sequence identity, and some demonstrate bimodal distribution where the two modes are well separated (e.g., *Circoviridae*, *Geminiviridae*, *Smacoviridae*, and pCRESS6). It is interesting to note that sequences like *Geminiviridae*, *Circoviridae*, and *Smacoviridae* exhibit narrow distribution in their length yet exhibit broad and multimodal distribution in their sequence identity. In contrast, sequences like CRESSV1b, CRESSV2, CRESSV3 and pCRESS4 exhibit broad distributions in length yet narrow distributions in sequence identity (Figure 1b). We performed an all-vs-all ANOVA Tukey test to determine if the difference of means between groups is statistically significant (Figure 1c). Indeed, the *p*-values attained from this analysis indicate that there is a statistically significantly distinction between most groups. Our analysis demonstrates that the length and identity of Rep sequences vary considerably within and between groups.

### 3.2. Tertiary Structure and Sequence Diversity among Reps

The PCV2 Rep polypeptide encodes for the three functionally and structurally distinct endonuclease, oligomerization, and ATPase domains [20,28,29,30,40,41]. We ask if all Reps possess comparable structures, and if the functionally important amino acids identified in the PCV2 Rep structure are also present in other Reps? To address whether all Reps possessed comparable structures, we used the recently developed TrRosetta to predict the structure of representatives for each of the 22 groups [35]. We mapped the sequence alignment from each group onto its predicted structure, compared each prediction to the experimentally determined structure of PCV2 Rep, and determined how well functionally important amino acids were conserved within each group. These analyses were preformed prior to the deposition of our structure into the protein data bank; thus, the predictions are not biased by the PCV2 OD-AD structures.

TrRosetta predicted a minimum of three domains for representatives from each group. To objectively determine if the predicted domains were homologous to the ED, OD, and AD of PCV2 Rep, we used root mean square deviation (rmsd) and TM-scores (topological similarity) calculated from structure superposition of the predicted and experimentally determined structures [20,28]. Structures with similar folds, defined by SCOP/CATH, exhibit TM-scores greater than 0.5 [36]. Structures with TM-scores greater than 0.5 may be proposed to be homologous (i.e., share a common ancestor). We then performed sequence alignment for each group, calculated a conservation score for each amino acid within the alignment, mapped the conservation scores onto the predicted structure, and converted these values to heat maps for visualization. The advantage of these maps, over conventional sequence alignment, is that they depict how amino acids positioned distantly to one another in a 1D sequence can be juxtaposed in a 3D structure. The juxtaposition of conserved amino acids identifies structurally and functionally important sites. We further extracted the sequences pertaining to the N-terminus, ED, OD, AD, and C-terminus for sequence, structure, and functional analysis.

#### 3.2.1. The Endonuclease Domain (ED)

**Figure 2:**
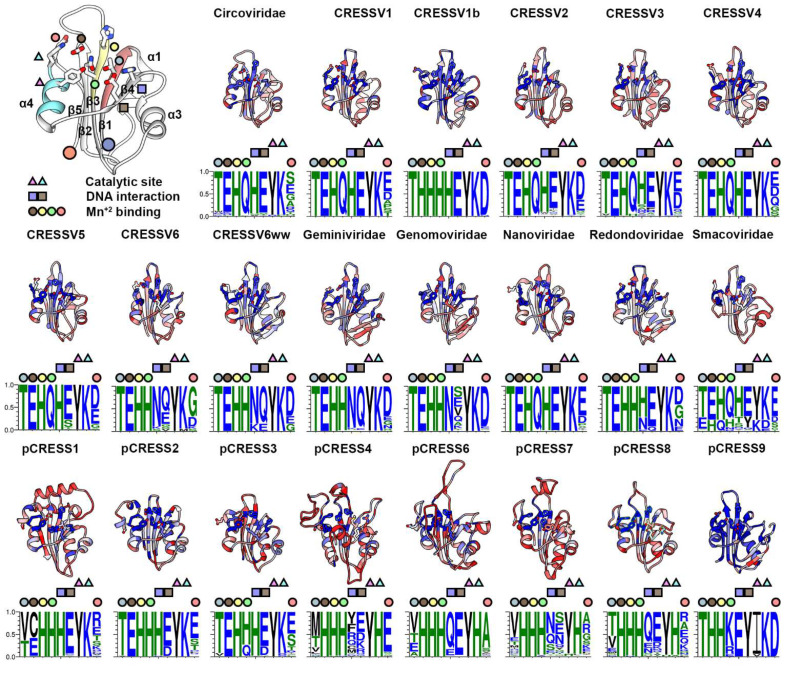
Tertiary structure comparison of CRESS-DNA and pCRESS-DNA Rep endonuclease domain (ED). Top left, ribbon cartoon of the PCV2 ED (PDB entry 5XOR). Motifs I–III are colored red, yellow and cyan. The secondary structure elements are labeled. Functionally important amino acids defining the catalytic site, the DNA interaction site, and the Mn^+2^ binding site are shown as stick figures and identified by triangles, squares and circles for clarity. Ribbon cartoons for each member of the 22 groups are colored using a heat map for least conserved (red) to most conserved (blue). Below each ribbon cartoon is a WebLogo image demonstrating the conservation of the indicated amino acids.

The rmsd values from the superposition of the predicted and experimentally determined structures vary from 0.5 Å (*Circoviridae*) to 3.4 Å (*Geminiviridae*), and the TM-scores vary from 0.6 (pCRESS6) to 0.9 (*Circoviridae*) (Table S1). These values indicate the 22 predicted ED structures to be potentially homologous to the PCV2 ED (Table S1, Figure 2). We then asked how conserved are the ED primary structures within each of the 22 groups? The distribution for ED length is wide for CRESSV1b, *Smacoviridae*, pCRESS1, and pCRESS4, whereas the distribution is narrow for pCRESS9, pCRESS2, *Redondoviridae*, and *Geminiviridae*, (Figure S1a). The *Smacoviridae* have the shortest ED (mean length of 78) while pCRESS6 have the longest ED (mean length of 142). ANOVA with Tukey test demonstrates that the difference of mean length between EDs is statistically significant (*p*-value < 0.05) (Figure S1a). The ED lengths of *Smacoviridae*, pCRESS1, pCRESS4 and pCRESS6 are significantly different than those from other groups. We then asked if the frequency of insertions is greater between particular β-strands. While insertions of five or more amino acids can be observed between all consecutive β-strands, there is clearly a bias for insertions between β1–β2 (14/22 groups), between β3–β4 (6/22), and between β2–β3 (4/22). Some of these insertions are more than 20 amino acids. The insertions between β1–β2 results in lengthening of α1. The insertion between β3–β4 form a β-sheet with a strand predicted at the N-terminus of CRESSV6ww, *Geminiviridae*, *Genomoviridae*, *Redondovirudae*, and pCRESS9 (Figure 2). The insertion between β3–β4 forms a two stranded β-sheet in the predicted structures of pCRESS4 and pCRESS6, a helix in pCRESS7, and a loop in pCRESS8. Further studies are needed to assess if these insertions play a role in regulation of ED.

Surprisingly, the distribution of ED sequence identity within each group is very broad (Figure S2a). CRESSV5 exhibits one of the narrowest distributions (30–89%). Conversely, *Geminiviridae* (22–94%), *Smacoviridae* (9–97%), and pCRESS1 (13–95%) exhibit the widest distribution (Figure S2a). ANOVA with Tukey test demonstrates that the difference in mean for sequence identity is statistically different between several groups, with the means of CRESSV1b, *Geminiviridae*, and pCRESS9 distinct from the remaining groups. The *Geminiviridae*, *Genomoviridae*, *Redondoviridae*, *Smacoviridae*, pCRESS1, pCRESS3, pRECC6, pCRESS7 and pCRESS9 demonstrate multimodal distribution of sequence identity. CRESSV1b (mean of 66%) and pCRESS9 (mean of 82%) possess the most conserved sequences. Having demonstrated that the ED sequence lengths and identity vary significantly within and between groups, we asked if groups that exhibit a wider distribution of lengths also exhibit a wider distribution of sequence identity? We answered this question by plotting the standard deviation in length for each group against the standard deviation of sequence identity for that group. The plots reveal no correlation (data not shown).

Sequence alignment studies of ED identified three conserved motifs (I–III) that were proposed as responsible for three distinct functions of ED (Figure 2). Motif I had been proposed to be involved in DNA recognition, Motif II had been proposed to be involved in coordination of a divalent metal ion (Mg^+2^ or Mn^+2^), and Motif III had been proposed to be involved in the DNA nick-ligase activity of ED [19,24]. Motifs I-III map to β1, β3 and α4 (Figure 2). Recent crystal structures of PCV2 and Wheat dwarf virus (WDV, a *geminiviridae*) ED in complex with the stem loop nonanucleotide ssDNA revealed the position of the nucleophilic Tyr with respect to its ssDNA substrate, the importance of a His/Glu/Gln (PCV2) or His/Glu/His (WDV) triad in binding a divalent cation (Mn^+2^) juxtaposed to the phosphate of the cleaved ssDNA substrate, and identification of a stretch of 9–10 amino acids (β4) responsible for defining the single-stranded DNA binding bridge motif (sDBM) [29]. The crystal structures demonstrate that Motif I amino acids exhibit limited interaction with the ssDNA, and thus disagree with the sequence-based predictions. Rather, the Motif I Thr is responsible for properly positioning the Motif II amino acids to coordinate the Mn^+2^. In Motif II, the side chains of Gln and His (PCV2 amino acids) or side chains of His and His (WVD amino acids) provide two binding partners for Mn^+2^. For PCV2, the Glu side chain (β2) provides the third binding partner, and a water molecule bound to the side chain of Glu (α4) provides the fourth binding partner. For WVD, a Glu in α4 provides the third and fourth binding partners for Mn^+2^. For both PCV2 and WVD Reps, the phosphates from the ssDNA provide the fifth binding partner for Mn^+2^. These geometries are congruent with quantum mechanical calculations as well as a recent survey of the PDB that demonstrated Mn^+2^ to exhibit a coordinate number of five to six [42]. Interestingly, WVD possesses the Glu present in the PCV2 β2, and PCV2 possesses the Glu present in WVD α4; thus, why do the two proteins use different Mn^+2^ binding partners? Surprisingly, the *Geminiviridae* Glu in α4 is not highly conserved (Figure 2). Indeed, this amino acid is poorly conserved among all CRESS-DNA Reps, with the exception of CRESSV1b. Thus, we hypothesize that the two ligands from β3 and any ligand from β2 or α4 is sufficient to properly coordinate Mn^+2^ for it to sufficiently delocalize the electrons of the scissile phosphate for a nucleophilic attack by the Tyr nucleophile. Only additional structural studies of Rep can answer this hypothesis. Tompkins et al. demonstrated that swapping a ten amino acid segment (β4) between PCV2 and WVD resulted in a change of preference for ssDNA substrate. Consequently, they named this region the single-stranded DNA binding motif (sDBM) [29]. However, only the side chains from two sDBM amino acids interact with the nucleotide bases of the ssDNA substrate (Figure 2). Consequently, it is these two amino acids that are likely responsible for defining the sDBM. Our heat maps reveal that while these amino acids are highly conserved, they are not absolutely conserved in CRESSV2, CRESSV3, CRESSV5, CRESSV6, CRESSV6ww, *Geminiviridae*, *Genomoviridae*, *Redondoviridae*, *Smacoviridae*, pCRESS2, pCRESS3, pCRESS4, pCRESS6, pCRESS7, and pCRESS8. Thus, it remains to be seen if additional factors in these Reps may provide an alternative sDBM. The Thr in Motif II is nearly absolutely conserved among all viral Reps, yet poorly conserved among pCRESS Reps (Figure 2). Finally, Kazlauskas et al. demonstrated that pCRESS4-8 possessed a YLxH signature in motif III (where x is any amino acid). Inspection of our heat maps and WebLogo images indicates that the Tyr is the nucleophile, and that the His is replaced by Lys in other Reps. The position of this amino acid and its conservation is likely to be important for catalysis. His/Lys may act as a general base to deprotonate the nucleophile Tyr for S_N_2 attack of the ssDNA.

#### 3.2.2. The Oligomerization Domain (OD)

**Figure 3:**
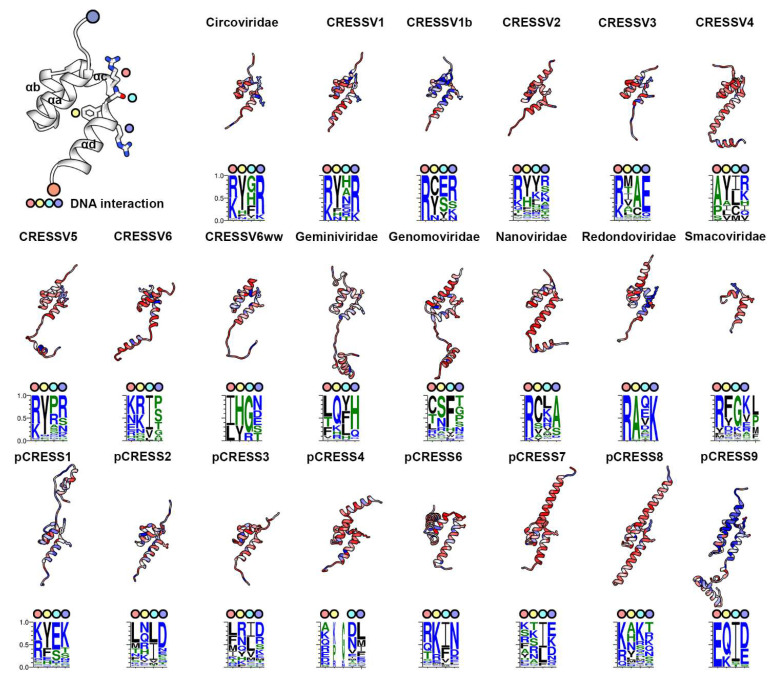
Tertiary structure comparison of CRESS-DNA and pCRESS-DNA Rep oligomerization domain (OD). Top left, ribbon cartoon of the PCV2 OD (PDB entry 7LAR). Potentially important amino acids that may interact with the translocating ssDNA are shown as stick figures, and further identified by colored circles. Ribbon cartoons for each member of the 22 groups are colored using a heat map for least conserved (red) to most conserved (blue). Below each ribbon cartoon is a WebLogo image demonstrating the conservation of the indicated amino acids.

The PCV2 OD is 60 amino acids long and folds into a four-helix bundle (αa-αd) (Figure 3). Three of these helices (αa, αb, and αd) are nearly parallel to one another, and the fourth (αc) is perpendicular to them. The OD is responsible for forming the hexameric Rep [20]. Oligomers are made by the αa-αd helices packing against the αb-αc helices of neighboring subunit (Figure 3). The rmsd values from the superpositions of the predicted structures onto the PCV2 experimental structure vary from 2.2 Å for CRESSV3 to 3.7 Å for pCRESS6, and the TM-scores vary from 0.4 for CRESSV6 to 0.7 for *Circoviridae* (Table S1). The OD structures for CRESSV6, pCRESS6 and pCRESS7 have TM-scores less than 0.5, suggesting that they may not be topologically similar to the experimentally determined PCV2 OD. However, visual inspection of the models demonstrates that they are indeed comparable (Figure 3). Consequently, the 22 predicted OD structures are topologically similar to the PCV2 OD and are likely to be homologous to one another (Table S1, Figure 3). The *Smacoviridae* OD is rather small and missing two of the helices (αb and αc) responsible for forming the hexameric OD pore. Thus, it remains to be seen if the *Smacoviridae* Rep indeed does form a hexamer. Surprisingly, the distribution of length within certain groups is quite large. CRESSV2, CRESSV6, *Genomoviridae*, and pCRESS7 exhibit wide distributions, while CRESSV1b, *Smacoviridae*, pCRESS2, pCRESS3, pCRESS6, and pCRESS9 exhibit narrow distributions (Figure S1b). The *Smacoviridae* have the shortest OD (mean length of 31) while pCRESS9 have the longest OD (mean length of 120). ANOVA with Tukey test demonstrates that the difference of mean length for OD is significantly different between groups (Figure S1b). CRESSV3, *Nanoviridae*, *Smacoviridae*, and pCRESS9 have lengths that are significantly different than the others. Additions to the termini of the OD core is observed for the CRESSV4, CRESSV5, CRESSV6, CRESSV6ww, *Geminiviridae*, *Genomoviridae*, and pCRESS4-9 (Figure 3).

The OD sequence identity within groups generally exhibit broad distributions (Figure S2b). CRESSV4 exhibits one of the narrowest distributions, with the majority of entries sharing 15–30% sequence identity. Conversely, *Smacoviridae* exhibit one of the widest distributions, with the majority sharing 5–60% sequence identity. ANOVA with Tukey test demonstrates that the difference between means is statistically significant between several groups. CRESSV1b, pCRESS3, and pCRESS9 are significantly different than the remaining 21 groups (Figure S2b). CRESSV1b, *Geminiviridae*, *Nanoviridae*, *Smacoviridae*, pCRESS2, pCRESS3, pCRESS6, and pCRESS9 exhibit bimodal distributions. Except for pCRESS3, the sequence diversity for OD is greater than the ED (Figure S2a,b).

The OD structure is homologous to that of other viral SF3 helicases whose structures have been determined that include: the papillomavirus E1, the SV40 large T antigen (LTag), the polyomavirus JCV helicase, and the adeno-associated virus 2 Rep68 [43,44,45,46]. Moreover, it is homologous to the OD of pMV158 replication initiator RepB [47]. With the exception of Rep68, all of these structures only form hexamers. Analysis of the OD–OD interfaces using the PDBePISA server demonstrates a diverse range of interactions that include: 440–730 Å^2^ of buried surface area, 0–7 hydrogen bonds, 0–7 salt-bridges, and no disulfide bonds [48]. In parallel to this, Rep68 seems to form both hexamers and heptamers. Unfortunately, the cryo-EM maps for the Rep68 hexamers and heptamers are of insufficient resolution to model the Rep68 side chains [46]. Thus, it remains to be discovered how diverse the Rep68 OD-OD interfaces can be.

The linker between the ED (β5) and OD (αa) has been proposed to be highly flexible, and this flexibility has been proposed to be responsible for the proper function of ED in binding to the *ori* [47]. We asked the question: how conserved is the length of this linker? Given the short length of *Smacoviridae* OD, we could not identify an ED–OD linker that would be equivalent to the other groups (Figure S3a). The *Nanoviridae* exhibit the shortest linker (4 amino acids), whereas the pCRESS6 exhibit the longest linker (27 amino acids). The variation in linker lengths for *Nanoviridae*, pCRESS9, and CRESSV1b is very limited, whereas for pCRESS8, pCRESS7, and CRESSV6 it is significant. ANOVA with Tukey test demonstrates that the difference of mean linker length is statistically significant between most groups (Figure S3b).

To determine if the PCV2 OD fold was topologically similar to other proteins in the PDB, we used the Dali and PDBeFold servers [48,49]. Matches include a pheromone (PDB entry 6E6N), Zn^+2^ binding proteins from retrovirus integrases (PDB entries 5CZ2, 6RWO, 1WJE, 2VXD, 3JCA), DNA binding domains of transcription factors (PDB entries 6DFY, 3A03), the RED subdomain of Sleeping Beauty transposase (PDB entry 5UNK), and DIP2311 from Corynebacterium diphtheriae, a Northeastern Structural Genomics target with an unknown function (PDB entry 3LMM). With the exception of the Structural Genomics target, these are homeodomain proteins. Homeodomain proteins are responsible for binding to DNA for regulation of transcription [50]. They accomplish this by binding to and expanding the major groove of dsDNA. Overlaying the dsDNA bound homeodomains onto the PCV2 Rep OD does not position the dsDNA into the pore defined by the six OD; however, homeodomains employ many distinct binding modes to DNA [50].

Consequently, we asked how conserved are the four amino acids that line the surface of the PCV2 OD hexameric pore since these amino acids may be responsible for interacting with the ssDNA that translocate through Rep during genome replication? To answer this question, we looked at the heat maps generated from the amino acid conservation described above (Figure 3). The predominantly red and white colors reflect the poor sequence conservation of the OD (Figure 3). The WebLogo images suggest that, for most groups, the first amino acid is either an Arg/Lys (*Circoviridae*, CRESSV1, CRESSV1b, CRESSV2, CRESSV3, CRESSV5, CRESSV6, *Nanoviridae*, *Redondoviridae*, *Smacoviridae*, pCRESS1, pCRESS6, and pCRESS8). The fourth amino acid is also conserved in some of the groups as either an Arg/Lys (*Circovirudae*, CRESSV1, CRESSV1b, CRESSV4, CRESSV5, *Redondoviridae*, and pCRESS1). The second and third amino acids are less conserved, varying between hydrophobic or hydrophilic. Groups with OD larger than PCV2 have additional amino acids at the C-termini of αD (CRESSV4, CRESSV5, CRESSV6, CRESSV6w, *Geminiviridae*, *Genomoviridae*, *Nanoviridae*, and pCRESS9).

#### 3.2.3. The ATPase Domain (AD)

**Figure 4:**
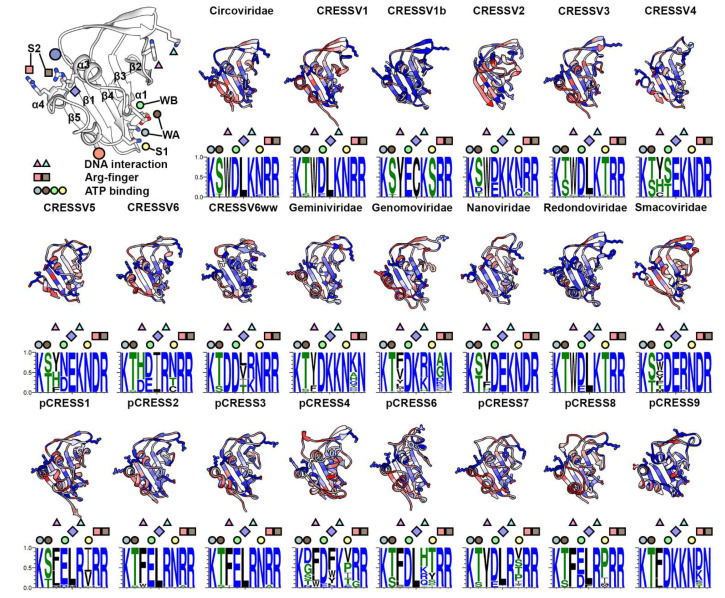
Tertiary structure comparison of CRESS-DNA and pCRESS-DNA Rep ATPase domain (AD). Top left, ribbon cartoon of the PCV2 AD (PDB entry 7LAR). Functionally important amino acids defining the DNA interaction site, Arg-finger, and ATP binding are shown as stick figures and identified by triangles, squares, and circles for clarity. Ribbon cartoons for each member of the 22 groups are colored using a heat map for least conserved (red) to most conserved (blue). Below each ribbon cartoon is a WebLogo image demonstrating the conservation of the indicated amino acids.

The rmsd values from the superpositions of the predicted structures onto the PCV2 experimental structure vary from 1.1 Å for *Circoviridae* to 3.1 Å for pCRESS6, and the TM-scores vary from 0.6 for pCRESS9 to 0.9 for *Circoviridae* (Table S1). Consequently, the 22 predicted AD structures are topologically similar to the PCV2 AD and are likely to be homologous to one another (Figure 4). CRESSV6ww exhibits the shortest AD (mean of 100), and pCRESS4 exhibits the longest AD (mean of 146). CRESSV1b and pCRESS2 exhibit the narrowest while *Genomoviridae* exhibits the broadest length distribution (Figure S1). ANOVA with Tukey test demonstrates that, except for *Geminiviridae*, *Redondoviridae*, pCRESS4, and pCRESS8, the mean lengths of viral AD are comparable. Given the significant differences of AD lengths within each group, we asked if insertions of amino acid occur at particular locations of AD? While insertions of five or more amino acids can be observed between all consecutive β-strands, there is a bias for these modifications to occur between β1–β2 (9/22), and β4–β5 (8/22). The insertions between β4–β5 are more than 20 amino acids. Plotting the standard deviation in length against the standard deviation in identity for each group does not reveal any correlation (data not shown).

The ADs exhibit broad distributions for sequence identity (Figure S2). However, means for sequence identity are consistently greater than those of the ED and OD. CRESSV6ww, CRESSV4, *Redondovirida*, and pCRESS2 exhibit the narrowest distribution. Conversely, *Geminiviridae* and *Smacoviridae* exhibit the widest distributions. ANOVA with Tukey test demonstrates that the difference in mean is statistically significant between several groups, with the means of CRESSV1b, *Geminiviridae*, and pCRESS9 significantly different than the remaining 21 groups (Figure S2). The *Circoviridae*, CRESSV6, *Geminiviri-dae*, *Smacoviridae*, pCRESS6 and pCRESS9 exhibit bimodal distributions. Insertions of five or more amino acids can be observed between every two consecutive β-strands; however, most insertions occur between β1–β2 (9/22), and β4–β5 (8/22). Some of these insertions are more than 20 amino acids.

Our structure of the PCV2 Rep hexamer bound to ADP and ssDNA confirmed the predicted nucleotide binding site of CRESS-DNA Reps, defined the Rep-ssDNA binding interface, and provided a mechanism for ssDNA translocation during genome replication [20]. To explore if these findings are conserved among CRESS-DNA Reps and their bacterial homologues, we explored the conservation of the amino acids responsible for these functions. The nucleotide binding site for SF3 ATPase members is formed by two neighboring subunits. The subunit that interacts most with the nucleotide provides the *cis*-components Walker A (WA, located between β1 and α1), Walker B (WB, located between β3 and α3), and sensor 1 (S1, following β4). The neighboring subunit provides the *trans*-component sensor 2 (S2, located in α4) [51]. WA binds to the β-phosphate of the nucleotide and is essential to its binding. WB co-ordinates the nucleotide’s Mg^+2^ and is essential for ATP-hydrolysis. S1 co-ordinates a water molecule for hydrolysis of the ATP γ-phosphate. S2 (Arg-finger) stabilizes the γ- and β-phosphates of the nucleotide pre- and post-hydrolysis. The heat maps and WebLogo images demonstrate that the Lys of WA is absolutely conserved, while the Ser can be exchanged to the comparable Thr. Interestingly, members of pCRESS4 possesses Asp/Gly/Asn at this position. WB is absolutely conserved as Asp in *Circoviridae*, CRESSV3, CRESSV6ww, *Geminiviridae*, *Genomoviridae*, *Nanoviridae*, *Smacoviridae*, pCRESS6, and pCRESS9. It is substituted to the comparable Glu in CRESSV1b, pCRESS1, and pCRESS2. Surprisingly, it is either Ser/Thr in CRESSV4 or Asp/Asn in CRESSV5. It would be interesting to determine if the WA Asp/Gly/Asn (pCRESS4) and WB Ser/Thr (CRESSV4) or Asn (CRESSV5) can hydrolyze ATP given that comparable substitutions in other ATPases have resulted in variants with diminished ATPase activity [52]. S1 is absolutely conserved as Asn or substituted to the comparable Gln in most groups. It is substituted to Thr/Ser/Cys (all of which can co-ordinate water) in CRESSV1b, CRESSV3, CRESSV6, and *Redondoviridae*. However, members of pCRESS1, pCRESS4, pCRESS6, pCRESS7, pCRESS8 and pCRESS9 possess Val/Pro/Ile/Ala/Met whose side chains are unable to hydrogen bond with the nucleophilic water molecule responsible for ATP hydrolysis (Figure 4). It remains to be determined if other amino acids in the vicinity of the ATP binding site may properly position the nucleophilic water. The S2 Arg-fingers are absolutely conserved among eleven of the groups. However, only the second Arg is conserved for CRESSV2, CRESSV3, CRESSV4, CRESSV5, *Nanoviridae*, *Smacoviridae*, pCRESS2, pCRESS3, and pCRESS4. Furthermore, only the second position is conserved for *Geminiviridae*, *Genomoviridae*, and pCRESS9, but as an Asn. The lack of two Arg is surprising as both are involved in interacting with and neutralizing the negative charges of the ADP α- and β-phosphates [20]. It remains to be seen how the *Geminiviridae*, *Genomoviridae*, and pCRESS9 Reps interact with the bound nucleotides.

Our Rep-ssDNA structure demonstrated that each subunit provided side chains from two amino acids to interact with the backbone of ssDNA [20]. The first involved Van der Waals interaction between the indole of a Trp (located in the β2 and β3 loop) and ssDNA ribose. The second involved electrostatic interaction between the amine of the pre-sensor 1 β-hairpin Lys (loop between α3 and β4) with the negatively charged phosphates of ssDNA (Figure 4). These two amino acids are absolutely conserved in most groups. In some groups the Trp has been substituted to another large amino acid (Tyr/Phe/His/Leu), which may function comparably. Surprisingly, members from the CRESSV4, CRESSV6, CRESSV6ww, and *Smacoviridae* possess either a small amino acid or an acidic amino acid at this position, properties that suggest an alternative mode of interaction with ssDNA. It remains to be determined how these Reps interact with ssDNA. The Lys is also highly conserved, sometimes substituted to the comparable Arg, except for pCRESS6. Some members of pCRESS6 possess a Gln at this position. Thus, it remains to be determined if members of pCRESS6 do indeed interact comparably with the ssDNA during its translocation. Our heat maps identified that, except for CRESSV6ww and pCRESS4, an additional amino acid is absolutely conserved in α3. *Circoviridae*, CRESSV1, CRESSV3, *Redondoviridae*, pCRESS1-3, and pCRESS6-7 possess a Leu. CRESSV2, *Geminiviridae*, *Genomoviridae*, and pCRESS9 possess a Lys. CRESSV4-5, *Nanoviridae*, and *Smacoviridae* possess a Glu. CRESSV1b possess a Cys, and CRESSV6 possess Ile. CRESSV6ww and pCRESS4 possess branched or hydrophobic amino acids at this position. The conservation of this position and its juxtaposition to the ATP binding site suggests that it may play an important role in the function of Rep.

#### 3.2.4. Extensions of the Termini

Mapping our sequence alignment onto the predicted structures demonstrated that the number of amino acids at the N- and C-termini of Rep was different within and between groups. Our analysis demonstrates that the lengths of the N-termini vary considerably within CRESSV2, CRESSV5, and pCRESS4 (Figure S4a). Similarly, the lengths of the C-termini vary considerably in CRESSV1b, CRESSV2, CRESSV3, pCRESS4, and pCRESS6 (Figure S4b). To determine if the difference of means is statistically significant, we used ANOVA with Tukey test (Figure S4c). The N-terminus mean length for pCRESS4 and the C-termini of pCRESS4, pCRESS6, and pCRESS7 are significantly larger than the remaining 21 groups.

Can structures predicted for these termini provide potential functional roles for the Rep? We predicted structures for representatives from groups whose termini mean size is greater than 50 amino acids. Representatives that satisfied these conditions were pCRESS4 (N- and C-termini), CRESSV1b (C-terminus), and pCRESS6 (C-terminus). We clustered the sequences for each group termini into groups sharing more than 30% sequence identity using the CD-Hit server [32]. We then predicted the structure of the three most populated sub-groups from each group. For structure prediction, three sequences were selected for the pCRESS4 group N-terminus, two sequences for the CRESSV1b C-terminus, and three sequences for the C-termini of each pCRESS4 and pCRESS6. The predicted structures of pCRESS4 N-terminus (GenBank WP_191397458.1), and pCRESS4 C-terminus (ERI66470.1, CEI31812.1, HIW92426.1) are globular, whereas the remaining predictions are non-globular. We submitted the predictions to the Dali server for the identification of potential homologues. Visual examination of the top hits provided by the Dali search confirmed potential homologues for two sequences: the pCRESS4 N- (WP_191397458.1) and C-terminus (ERI66470.1) The top two Dali hits for the N-terminus (Z-scores of 7.7 and 6.2) are a Denovo protein design by the Rosetta group (PDB 6MRS) and the C-terminal fragment of Gsp (PDB 5HL8) -a constituent of the Pululanase-specific Type II secretion system [53]. The top two hits for the C-terminus (Z-scores of 3.7 and 3.3) are the negative regulatory domain (NRD) of FOXM1 transcription factor (PDB 6OSW) and the C-terminus of PAAK Family AMP-Ligase [54,55]. NRD are responsible for binding and inhibiting the transactivation domain (TAD) of certain transcriptional machinery. TADs are responsible for recruiting machinery to target promoters [56]. It remains to be determined if the C-terminus of pCRESS4 serves a similar function.

## 4. Discussion

More than half a century ago Gilbert and Dressler described the molecular mechanism of genome replication by bacteriophage φX174 as RCR [1]. RCR has been reported to be used by members from all three domains of life, and the viruses that infect them [19]. Most studies have focused on determining the molecular mechanism of RCR, while few have assessed the structural mechanism. The Reps encoded by CRESS-DNA viruses are responsible for initiating RCR of the viral genome. These viruses do not encode for a DNA polymerase and are thus dependent on the cellular replication machinery for RCR. It remains to be described how and which cellular machinery are recruited to the viral *ori* for genome replication. The ED of Rep recognizes and binds to *ori* [14]. The NMR and crystal structures of the unliganded *Geminiviridae*, *Nanoviridae*, and PCV2 ED provided structural insight into the functionally important sites of this enzyme [28,30,40,41]. However, these studies did not determine the mechanism by which Rep recognized the *ori*. Upon binding *ori*, Rep is believed to alter the structure of the genome to generate a cruciform. It remains to be described if one or multiple Rep concurrently recognize *ori*, and how Rep drives cruciform formation. Following the formation of the cruciform, the ED of Rep recognizes a nonanucleotide sequence in the loop of the (+) strand DNA and hydrolyses this sequence at a specific site [14]. The crystal structures of the PCV2 and WVD EDs in complex with their respective nonanucleotides provided the mechanism of how ED recognize their ssDNA substrates [29]. ED hydrolyses the DNA using a Tyr nucleophile that becomes covalently attached to the 5′-PO_4_ ssDNA during the remaining steps of genome replication. The structure of Rep covalently attached to its hydrolytic product remains to be described. Following hydrolysis of the DNA, Rep assembles around the (-) ssDNA for DNA unwinding in a 5′-to-3′ direction. It remains to be described if it is the Rep that hydrolyzed the (+) strand DNA or a new Rep that is responsible for DNA unwinding. The cryo-EM structure of Rep bound to ssDNA and ADP provided the mechanism of how Rep interacts with the ssDNA during DNA unwinding [20]. Following replication of the template strand, ED hydrolyses the opposite end of the (+) strand DNA to liberate the ssDNA. ED then ligates the two ends of this ssDNA to generate the circular ssDNA genome. It remains to be described if one or multiple ED are responsible for the ligation reaction, and how ED recognizes the 3′- end that is to be ligated to the ED bound 5′-end. Furthermore, it remains to be discovered which and how the cellular machinery is recruited to the genome for RCR. Consequently, many structural studies need attention if we are to attain a better understanding of RCR. Our PCV2 Rep structure inspired us to ask the following questions regarding all CRESS-DNA and their bacterial homologues. How conserved are the primary and tertiary structures of Reps, how conserved are the functional centers of Reps, and are there distinctions between Reps that may shed light on their function?

Kazlauskas et al. employed sequence analysis and literature searches to identify 23 groups of CRESS-DNA Reps [19]. Fourteen of these were designated as viral CRESS Reps (CRESSV), and nine were designated as prokaryotic Reps (pCRESS) (Figure 1). We expanded the sequences identified by Kazlauskas et al. to include a total of 1595 sequences. We demonstrated that the sequence lengths for CRESSV1b, CRESSV2, CRESSV3, pCRESS4, and pCRESS6 are diverse, whereas the sequence identity for *Geminiviridae*, *Genomoviridae*, *Nanoviridae*, *Smacoviridae*, and pCRESS6 are diverse (Figure 1a,b). Consequently, no correlation exists between diversity in sequence length and identity. Moreover, the distribution of sequence length and identity are significantly different between many of the groups (Figure 1c). We used TrRosetta to predict the structures of Rep from a representative for each group. All representatives demonstrated a minimum of three domains. All groups exhibited both the ED and AD, and all but *Smacoviridae* exhibited comparable OD; the sequence length of *Smacoviridae* OD is too short for the four-helix bundle to form. A Dali search of the experimentally determined PCV2 OD demonstrated the fold to be comparable to the homeodomain. Homeodomains are responsible for binding dsDNA for regulating transcription [50]. Our structure of the PCV2 Rep suggested that the OD hexamer pore is the conduit through which ssDNA is translocated during genome replication [20]. Given the shared fold and function, it is likely that the OD and homeodomain share a common ancestor. We note that homeodomains have rarely been identified in prokaryotes [50,57].

We provide a three-dimensional interpretation of sequence conservation by mapping the amino acid conservation score from each group’s sequence alignments onto its representative structure (Figure 2, Figure 3 and Figure 4 and Figure S2). This representation clearly demonstrates that few amino acids other than the functionally important amino acids are conserved. Indeed, there is significant sequence diversity within each group for ED (Figure S2). Only the Thr of Motif I and Lys/His of Motif III are absolutely conserved (Figure 2). Thompkins et al. demonstrated that the Motif I Thr did not interact with the *ori* stem-loop nonanucleotide sequence, as had been predicted [29,58]. Rather, the Thr positions the neighboring His/Gln in Motif II to bind to the catalytically important Mn^+2^. The Lys/His present in Motif III (α4) is likely to act as the general base for deprotonating the Tyr nucleophile for attacking the ssDNA scissile bond. Interestingly, the lack of absolute conservation for the ssDNA binding bridge motif in CRESSV2, CRESSV3, CRESSV5, CRESSV6, CRESSV6ww, *Genomoviridae*, *Redondoviridae*, *Smacoviridae*, pCRESS4, pCRESS7 and pCRESS8 suggests that the binding mode of the nonanucleotide sequence to Rep may need further investigation. We also identify a number of insertions between the β1–β2 and β3–β4 strands of ED that may be functionally important. Further structural and biochemical studies are needed to address this possibility. For the OD, there is very little sequence conservation within each group (Figure 3 and Figure S2b). Our PCV2 Rep structure identified four amino acids that form the inner surface of the OD pore, and thus may be responsible for interacting with the ssDNA as it is translocated by Rep during dsDNA unwinding [20]. Our analysis demonstrates that the first and fourth amino acid for most groups is either an Arg/Lys, while the second and third vary between hydrophobic or hydrophilic amino acids. Nonetheless, it remains to be experimentally determined if these amino acids do indeed interact with nucleotides during ssDNA translocation. We determined that the connector between the ED and OD varies from 4 to 27 amino acids. Boer et al. had suggested that the linker was important for providing ED with the needed plasticity for DNA binding and manipulation. However, the short length of the *Nanoviridae* linker, the long length of the pCRESS6 linker, and the variable length of the CRESSV6 linker suggest that the exact role of this linker needs further structural and functional studies (Figure S3). For AD, the WB acidic amino acid is not absolutely conserved (Figure 3). CRESSV4, and CRESSV5 exhibit a Ser/Thr/Asn at this position. It has been shown that substitution of the WB to a non-acidic amino acid significantly diminishes in vitro ATPase activity [52]. Using the predicted structures, we were unsuccessful in identifying any neighboring acidic amino acids that could serve as the WB for coordinating the Mg^+2^. Thus, it would be interesting to determine if members are lacking the traditional WB hydrolyze ATP. The S1 amino acids are not absolutely conserved among ATPases, and amino acids able to coordinate the nucleophilic water molecule function without issues [52]. However, the Ile/Val/Pro/Ala/Met amino acids present in some members of the pCRESS1, pCRESS4, pCRESS6, pCRESS7, and pCRESS8 are insufficient to properly coordinate the water molecule (Figure 4). It would be interesting to determine if such Rep use an alternative mechanism to coordinate the nucleophilic water molecule. The ssDNA binding interface is made of a large hydrophobic amino acid that interacts with the ssDNA ribose, and a basic amino acid that interacts with the ssDNA phosphate (Figure 4). The large hydrophobic amino acid is not conserved in CRESSV4, *Genomoviridae*, *Smacoviridae*, pCRESS1, and pCRESS4, and the basic amino acid is either a His or Glu in pCRESS6 (Figure 4). It would be interesting to determine if such Rep use an alternative mechanism to interact with ssDNA for DNA translocation. We also identify a highly conserved amino acid within each group that is located in α3 and juxtaposed to the ATP binding site (Figure 4). The conservation and position of this amino acid suggest that it may be functionally important. It remains to be investigated if this amino acid plays an important role in the helicase activity of Rep. Finally, we identify members from pCRESS4 that possess extended N-termini, and members from CRESSV1b, pCRESS4, and pCRESS6 that possess extended C-termini. Structure prediction, DALI data base searches, and literature searches suggest that some members of the pCRESS4 may possess a negative regulatory domain at their C-termini. It would be interesting to determine experimentally if such domains do exist in pCRESS4. Consequently, there is tremendous opportunity for structural and biochemical studies of CRESS-DNA and pCRESS-DNA Reps to provide much needed insight into understanding the beauty of RCR.

## Data Availability

Sequences used in this study have been uploaded as supplementary data. The predicted structures have been uploaded to https://www.modelarchive.org/ with accession numbers: ma-0mufs, ma-fino4, ma-548ka, ma-tzmej, ma-ubwz5, ma-gd6vs, ma-yu4p0, ma-pzbd1, ma-eqgca, ma-sqz1w, ma-8lnf6, ma-zy441, ma-oogyg, ma-fxrza, ma-9oj1w, ma-ifxws, ma-tc8aw, ma-o7iss, ma-kctuz, ma-59b64, and ma-8cusv.

## Supplementary Materials

The following are available online at https://www.mdpi.com/article/10.3390/v14010037/s1, Figure S1: Primary structure analysis of Rep domains length, Figure S2: Primary structure analysis of Rep domain identity, Figure S3: Primary structure analysis of Rep ED-OD linker, Figure S4: Primary structure analysis of Rep termini, Table S1: Tertiary structure comparison of Rep domains.
