# Supplementary material for: A Structural Perspective of Reps from CRESS-DNA Viruses and Their Bacterial Plasmid Homologues
Source: Viruses. 2021 Dec 25;14(1):37. doi: 10.3390/v14010037 (PMC8780604; doi:10.3390/v14010037)
Supplement: Supplementary file 1 [file viruses-14-00037-s001.zip › manuscript_Replicase_Supplementary.pdf]

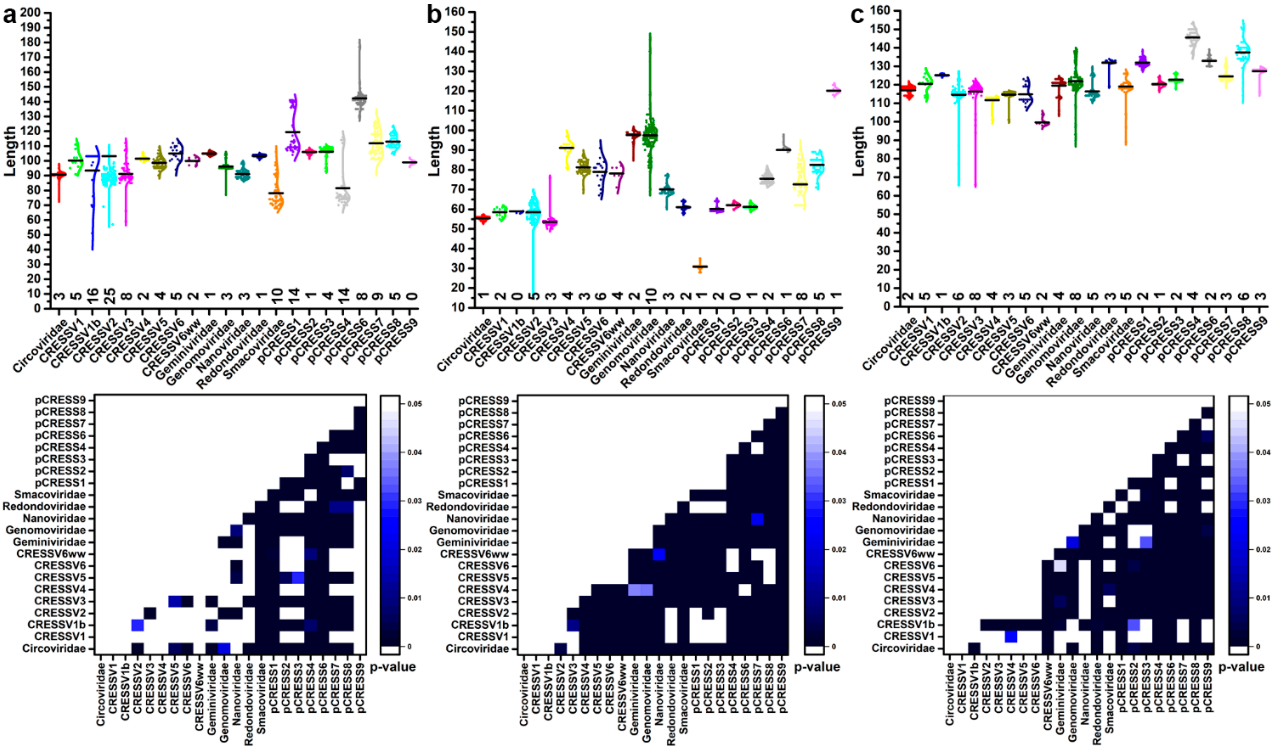

Figure S1. Primary sequence analysis of Rep domains for each family. (a) top, box scatter plot of Rep ED lengths from 1,595 sequences deposited in GenBank. The numbers at the bottom are the standard deviations. Bottom, heat map of p-values generated using ANOVA with Tukey analysis; (b) top, box scatter plot of Rep OD lengths. The numbers at the bottom are the standard deviations. Bottom, heat map of p-values generated using ANOVA with Tukey analysis; (c) top, box scatter plot of Rep AD lengths. The numbers at the bottom are the standard deviations. Bottom, heat map of p-values generated using ANOVA with Tukey analysis.

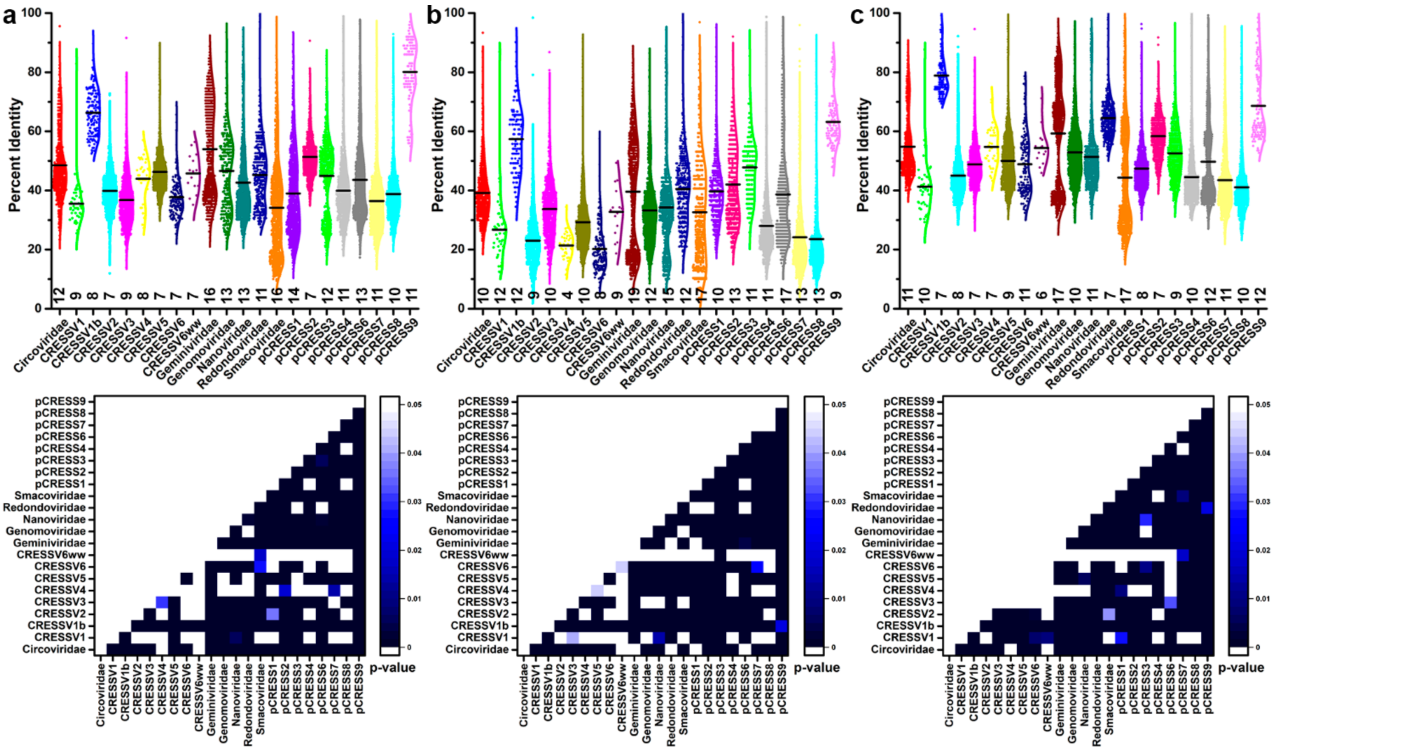

Figure S2. Primary sequence analysis of Rep domains for each family. (a) top, box scatter plot of Rep ED sequence identity from 1,595 sequences deposited in GenBank. The numbers at the bottom are the standard deviations. Bottom, heat map of p-values generated using ANOVA with Tukey analysis; (b) top, box scatter plot of Rep OD sequence identity. The numbers at the bottom are the standard deviations. Bottom, heat map of p-values generated using ANOVA with Tukey analysis; (c) top, box scatter plot of Rep AD sequence identity. The numbers at the bottom are the standard deviations. Bottom, heat map of p-values generated using ANOVA with Tukey analysis.

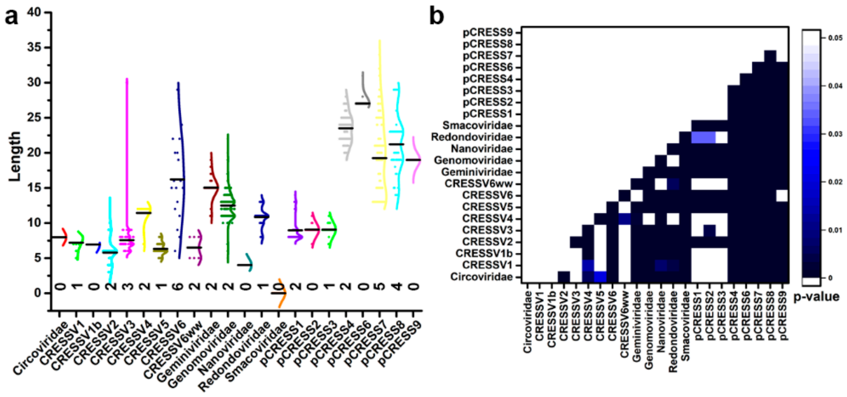

Figure S3. Primary sequence analysis of the Reps ED-OD linker for each family. (a) box scatter plot of ED-OD linker from 1,595 sequences deposited in GenBank. The numbers at the bottom are the standard deviations; (b) Heat map of p-values generated using ANOVA with Tukey analysis.

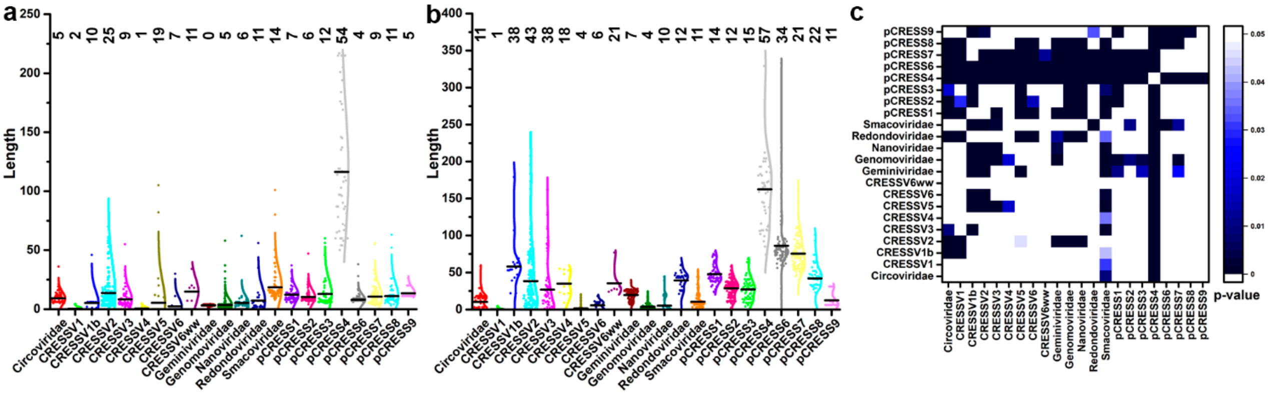

Figure S4. Primary sequence analysis of Rep N- and C-termini for each family. (a) Box scatter plot of Rep N-termini lengths. The numbers at the top are the standard deviations; (b) Box scatter plot of Rep C-termini lengths. The numbers at the top are the standard deviations; (c) Heat maps of p-values generated using ANOVA with Tukey analysis for N-terminus (bottom right) and top left C-terminus.

	ED			OD			AD		
**Sequence**	**rmsd**	**TM-score**	**ID**	**rmsd**	**TM-score**	**ID**	**rmsd**	**TM-score**	**ID**
CRESSV1	1.79	0.83	26	2.74	0.60	33	1.82	0.85	36
CRESSV1b	1.97	0.77	20	2.99	0.61	23	2.87	0.79	27
CRESSV2	2.59	0.82	34	2.99	0.60	13	2.21	0.71	34
CRESSV3	2.23	0.84	34	2.22	0.65	18	1.91	0.85	33
CRESSV4	2.20	0.82	41	2.25	0.54	7	2.59	0.76	15
CRESSV5	2.18	0.83	40	2.44	0.63	14	2.85	0.70	16
CRESSV6	3.20	0.69	18	2.74	0.42	9	1.93	0.83	23
CRESSV6ww	2.81	0.70	16	2.61	0.55	10	2.71	0.70	20
Circoviridae	0.52	0.94	100	2.47	0.68	100	1.05	0.87	100
Geminiviridae	3.40	0.63	21	2.72	0.56	18	2.35	0.70	13
Genomoviridae	3.19	0.70	16	2.67	0.53	17	2.3	0.78	16
Nanoviridae	2.44	0.79	29	2.86	0.59	12	2.74	0.70	14
Redondoviridae	3.13	0.67	13	3.18	0.50	10	2.50	0.70	30
Smacoviridae	2.61	0.71	23	2.32	0.50	0	2.39	0.78	17
pCRESS1	2.72	0.69	15	2.86	0.60	15	2.38	0.79	21
pCRESS2	2.32	0.74	25	2.59	0.52	5	1.93	0.79	23
pCRESS3	2.66	0.72	19	2.81	0.58	20	2.04	0.79	24
pCRESS4	2.65	0.70	10	2.64	0.58	17	2.46	0.78	20
pCRESS5	2.69	0.68	11	2.78	0.57	4	2.47	0.76	20
pCRESS6	3.17	0.63	11	3.72	0.45	6	3.10	0.73	21
pCRESS7	2.51	0.69	7	2.96	0.48	2	2.54	0.75	16
pCRESS8	2.76	0.71	15	2.67	0.59	9	2.21	0.77	21
pCRESS9	3.12	0.67	17	2.85	0.56	6	3.09	0.62	11

Table S1. Structural comparison of predicted Rep domains. Reported root mean standard deviation (rmsd), TM score, and sequence identity from structural superposition of the predicted structure onto the experimentally determined PCV2 (Circoviridae) ED (PDB entry: 5XOR) and OD-AD (PDB entry: 7LAR) structures using the program TM-align.
